# Oxytocin Signalling in Lower Urinary Tract Symptoms Associated with Benign Prostatic Hyperplasia and Overactive Bladder Syndrome: A Narrative Review

**DOI:** 10.3390/biomedicines14102306

**Published:** 2026-10-10

**Authors:** Masroor Badshah, Jibriil Ibrahim, Khaled A. Elnahriry, Ralf Middendorff, Michael Whittaker, Betty Exintaris

**Affiliations:** 1 Hudson Institute of Medical Research, Monash University, Clayton, VIC 3168, Australia; 2 Monash Institute of Pharmaceutical Sciences, Monash University, Parkville, VIC 3052, Australia; 3 Department of Pharmaceutical Sciences, College of Pharmacy, Alfaisal University, Riyadh 11533, Saudi Arabia; 4 Institute of Anatomy and Cell Biology, Justus-Liebig-University, 35390 Giessen, Germany

**Keywords:** lower urinary tract symptoms, benign prostatic hyperplasia, overactive bladder, oxytocin, oxytocin receptor, smooth muscle, atosiban, cligosiban, artificial intelligence, machine learning

## Abstract

Lower urinary tract symptoms (LUTSs) associated with benign prostatic disease and overactive bladder syndrome (OAB) substantially impair quality of life. This narrative review evaluates oxytocin receptor (OXTR) localisation, signalling, and contractile responses in prostate and bladder tissue, with anatomical and pharmacological context restricted to interpretation of these findings. Experimental receptor expression and antagonist responses provide mechanistic plausibility, but species differences, vasopressin receptor cross-reactivity, and inconsistent human tissue responses limit translation. Preclinical effects on tissue contractility and reproductive endpoints are distinguished from clinical urinary outcomes. OXTR antagonism remains investigational for LUTSs and OAB; clinical efficacy and safety require validation. Proposed artificial intelligence applications are limited to experimental receptor localisation, contraction analysis, and phenotyping.

## 1. Contractile Signalling Relevant to Oxytocin Research

Literature-search approach. PubMed, Scopus, Web of Science, and Google Scholar were searched, with the last search performed on 10 September 2026. Search terms included “oxytocin”, “oxytocin receptor”, “prostate”, “bladder”, “lower urinary tract symptoms”, “benign prostatic hyperplasia”, “overactive bladder”, “smooth muscle”, “atosiban”, and “cligosiban”, used in relevant combinations. Experimental studies, human tissue studies, clinical studies, reviews, and guidelines were selected for their relevance to oxytocin signalling, prostate and bladder contractility, and the anatomical or therapeutic context needed to interpret these findings. This was a narrative review, not a systematic review.

Oxytocin can activate G protein-coupled receptor signalling through phospholipase C and inositol trisphosphate, increasing intracellular Ca^2+^ and Ca^2+^–calmodulin-dependent myosin light chain kinase activity. Myosin phosphorylation and the balance between kinase and phosphatase activities determine contraction; Rho-kinase-associated pathways can increase Ca^2+^ sensitivity [1,2,3]. These shared mechanisms explain contractile plausibility but do not by themselves establish an OXTR-specific effect in prostate or bladder tissue.

Nitric oxide–cGMP signalling promotes relaxation by reducing intracellular Ca^2+^ availability and contractile Ca^2+^ sensitivity [4,5,6,7]. Adrenergic and muscarinic responses provide functional comparators when testing whether OXTR antagonists selectively inhibit oxytocin-mediated activity or suppress smooth muscle contraction more broadly [8,9,10,11,12,13,14,15,16,17].

Spontaneous prostatic activity has been linked to interstitial cells and resting myogenic tone [18,19,20,21]. Oxytocin increased spontaneous contraction frequency, and atosiban attenuated this response in experimental prostate preparations [22]. Such activity is an experimental endpoint and should be interpreted alongside nerve- and agonist-induced responses rather than assumed to represent clinical outlet obstruction.

## 2. Clinical Terminology and Interpretation of Tissue Responses

Male lower urinary tract symptoms (LUTSs) comprise storage, voiding, and post-micturition symptoms and describe a clinical presentation rather than a specific diagnosis [23,24]. Histological benign prostatic hyperplasia (BPH) denotes epithelial and stromal hyperplasia; benign prostatic enlargement (BPE) describes increased prostate size; and benign prostatic obstruction (BPO) denotes bladder outlet obstruction attributable to benign prostatic disease. Histological BPH, BPE, and BPO may coexist, but none should be inferred solely from the presence of another [25,26,27]. Prostate enlargement alone does not establish the cause of LUTS, which may reflect bladder, outlet, neurological, or other contributing disorders [28,29].

Overactive bladder (OAB) is a symptom-defined syndrome of urgency, usually with frequency and nocturia, with or without urgency urinary incontinence, in the absence of urinary tract infection or other obvious pathology. Detrusor overactivity (DO) is a urodynamic observation of involuntary detrusor contractions during filling cystometry [30]. OAB does not require demonstration of DO, and DO should not be used as a synonym for OAB. OAB may coexist with BPE or BPO but does not itself cause urethral obstruction. Receptor expression or contractility in prostate and bladder preparations does not establish any of these clinical diagnoses [31,32,33,34,35].

## 3. Tissue Context for OXTR Studies

Urine storage and emptying require coordinated detrusor contraction and outlet relaxation [36,37,38,39]. Reduced contraction in an isolated preparation cannot therefore be assumed to improve LUTSs: bladder inhibition may also compromise emptying. Tissue origin, cellular compartment, and experimental age are essential to interpreting OXTR localisation and antagonist effects.

### 3.1. Prostate Sampling and Cellular Compartments

The prostate surrounds the proximal urethra, and its smooth muscle contributes to outlet resistance and reproductive function [40,41,42,43]. Human zonal anatomy differs from the rat lobar organisation; BPH principally affects the transition/periurethral region [44,45]. Studies should report the sampled zone or lobe and stromal content when comparing receptor localisation and contractile responses across species [46,47,48,49,50,51,52,53,54]. Epithelial receptor staining should be distinguished from stromal localisation and from direct evidence of smooth muscle function.

### 3.2. Bladder Compartments and Tissue Maturity

The bladder wall contains urothelium, lamina propria, and detrusor smooth muscle [55,56,57]. Mucosal, neural, and interstitial influences can modify strip responses; studies should specify whether the mucosa is retained and identify the compartment used for receptor localisation. Prostate and bladder maturation also differ between species, so age and tissue maturity should be considered in model selection [58,59,60,61,62]. These developmental observations do not establish a role for OXTR in organ development.

## 4. BPH Context and Oxytocin Signalling

Histological BPH involves stromal and epithelial proliferation. BPE and increased smooth muscle tone may contribute to outlet resistance, but neither histological BPH nor BPE alone establishes BPO or explains male LUTSs [63,64]. Metabolic, inflammatory, and hormonal influences provide context for interpreting oxytocin experiments [65].

### 4.1. Metabolic and Inflammatory Context

Metabolic syndrome and its components have been associated with prostate growth and progression of benign prostatic disease [66,67,68,69,70,71,72,73]. Inflammation and interleukin/insulin-like growth factor signalling may also contribute to growth and sensory changes [74,75,76]. These associations identify relevant experimental covariates, particularly obesity and diabetes; they do not establish that OXTR mediates the clinical associations.

### 4.2. Steroid Context

Dihydrotestosterone and androgen receptors influence prostatic growth and secretion [77,78,79,80]. Oestrogen–androgen interactions may also affect prostatic biology [81,82,83]. Steroid conditions should be reported in oxytocin experiments because gonadal steroids can modulate prostatic oxytocin secretion and cellular responses [84,85].

### 4.3. Oxytocin and OXTR Evidence

Oxytocin (OT) is a hypothalamic nonapeptide released into the circulation [86,87]. Experimental studies in rabbit bladder have reported oxytocin-related detrusor contractile responses [88]. Tarhan et al. found no significant OT-induced contraction in human detrusor strips over 10^−9^–10^−5^ M (1 nM–10 µM), although one strip showed a slight response at a high concentration. Carbachol responsiveness was preserved. Atosiban was tested at 10^−5^ M (10 µM); its inhibition of OT-induced contractions was demonstrated in rat uterine controls, rather than established in human detrusor [89]. Species and experimental conditions therefore limit extrapolation to human bladder tissue.

OXTR is a seven-transmembrane G protein-coupled receptor [90,91]. Increased receptor expression has been reported in experimental and human hyperplastic prostate tissue [35]. Expression provides a biological rationale, but receptor localisation requires validation and does not demonstrate therapeutic benefit.

Oxytocin’s established roles in uterine contraction and milk ejection [92,93], together with its central and behavioural actions [94,95], provide context for possible systemic effects of OXTR antagonism. These functions are relevant to antagonist selectivity and reproductive safety rather than evidence of urinary efficacy.

Oxytocin is implicated in male reproductive tract smooth muscle regulation [96,97]. Gonadal steroids can modulate oxytocin secretion by human prostate tissue [84]. Prostatic neuroendocrine cells release paracrine mediators [98], but local cellular sources and receptor localisation require direct validation rather than inference from contractile activity.

**Figure 1:**
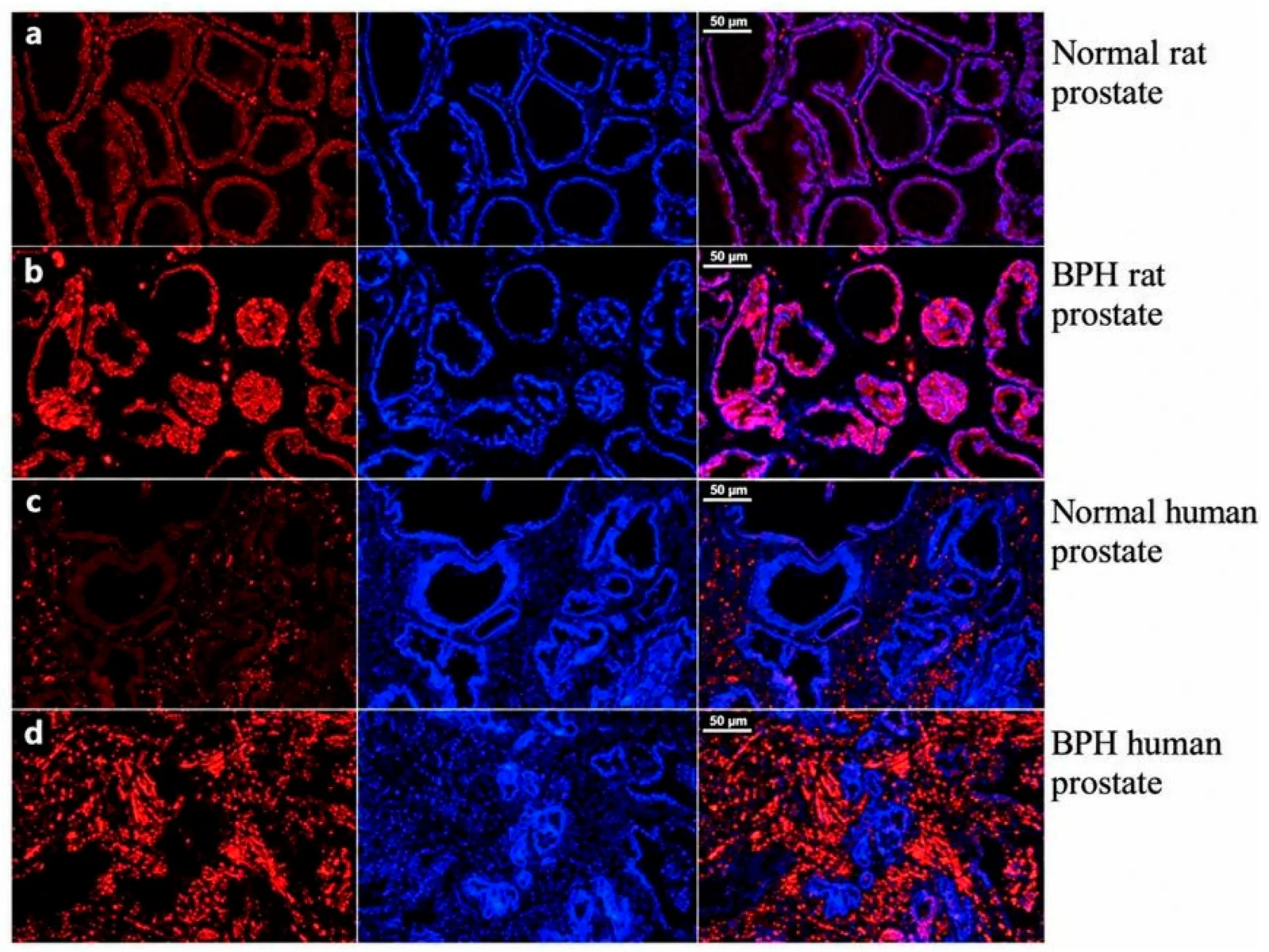
Localization of the oxytocin receptor (OXTR) in rat and human prostate tissue. The left column shows Cy3 immunofluorescence staining (red), indicating OXTR immunoreactivity; the middle column shows DAPI (4′,6-diamidino-2-phenylindole) nuclear staining (blue); and the right column presents the merged images (magnification ×50). (**a**) Normal rat prostate; (**b**) rat prostate with benign prostatic hyperplasia (BPH); (**c**) normal human prostate; and (**d**) human prostate with BPH. OXTR is predominantly localized in the prostatic epithelium in rats and in the prostatic stroma in humans. Reproduced with permission from Figure 8 of Li et al. [35].

Oxytocin-related prostatic contraction has been reported across experimental species [34]. Rat epithelial turnover and prostatic paracrine effects have also been investigated [99,100]. Human prostate proliferation findings should be interpreted according to cell type and steroid conditions [85,101]. OXTR localisation in epithelial and stromal compartments (Figure 1) provides a rationale for separating contractile effects from growth-related outcomes [35,91].

## 5. Bladder Mechanisms Relevant to Oxytocin Experiments

The mechanisms underlying symptom-defined OAB remain incompletely understood. Urothelial, myogenic, and neurogenic processes may contribute to urgency and other storage symptoms, and some may also contribute to urodynamically demonstrated DO. OAB and DO can overlap but describe different observations; OAB symptoms do not establish DO [30,102].

### 5.1. Urothelial and Myogenic Influences

Urothelial mediators, including acetylcholine and ATP, can influence afferent excitability and detrusor activity [103,104,105,106]. Mucosal injury, inflammation, and interstitial or gap-junction-associated mechanisms may modify spontaneous activity [107,108,109,110,111,112,113,114,115,116,117,118]. These factors should be considered when interpreting oxytocin responses, without attributing them to OXTR unless receptor involvement is demonstrated.

### 5.2. Neural Influences

Altered central inhibition and peripheral afferent activity can contribute to detrusor overactivity [119,120,121]. Isolated strips exclude much of this neural regulation, limiting extrapolation from oxytocin-induced contraction to bladder filling, voiding, and symptom-defined OAB.

## 6. Established Treatments and Investigational OXTR Antagonism

Management of male LUTSs and OAB provides the clinical context for evaluating investigational OXTR antagonists [28,29,102]. The label “LUTS/BPH” is retained only when used in the reported study; it does not establish histological BPH, BPE, or BPO.

### 6.1. Guideline-Based Management of Male LUTS

Guideline-based management considers symptom burden, likely contributing disorders, prostate size, progression risk, comorbidity, and patient preference. Conservative measures are appropriate for selected patients. α1-blockers provide symptom relief, while finasteride or dutasteride is used in men with BPE and increased progression risk; combination therapy is appropriate in selected men with enlargement and bothersome symptoms. Tadalafil 5 mg once daily is another established option for male LUTS, with or without erectile dysfunction. Selection should account for adverse effects, including blood-pressure and sexual effects [28,29].

For men with persistent storage symptoms, an antimuscarinic or β3-adrenoceptor agonist may be considered alone or with an α1-blocker, taking account of post-void residual urine and retention risk [28,29,102]. These established treatments provide the clinical context for studying OXTR antagonism; their benefits do not establish that experimental inhibition of tissue contraction by an OXTR antagonist will improve LUTSs.

### 6.2. Guideline-Based Management of OAB

OAB management follows shared decision-making rather than a mandatory treatment sequence. Behavioural measures and bladder training may accompany treatment. Antimuscarinics and β3-adrenoceptor agonists are alternative oral options, and may be combined when one medication provides inadequate relief. Selection considers cognitive risk, other adverse effects, contraindications, interactions, and patient preference. In men, possible BPO and impaired emptying require consideration; OAB treatment does not require demonstration of DO [28,29,102].

Intradetrusor botulinum toxin, percutaneous tibial nerve stimulation, and sacral neuromodulation are procedural options selected according to symptoms, preferences, response, and tolerability. Prior treatment trials are not mandatory in every case. Botulinum toxin counselling should include retention risk and possible intermittent catheterisation. Tadalafil’s established role in male LUTSs should not be extrapolated to routine treatment of idiopathic OAB [28,29,102].

### 6.3. Oxytocin Receptor Antagonists in Lower Urinary Tract Research

#### 6.3.1. Atosiban

**Figure 2:**
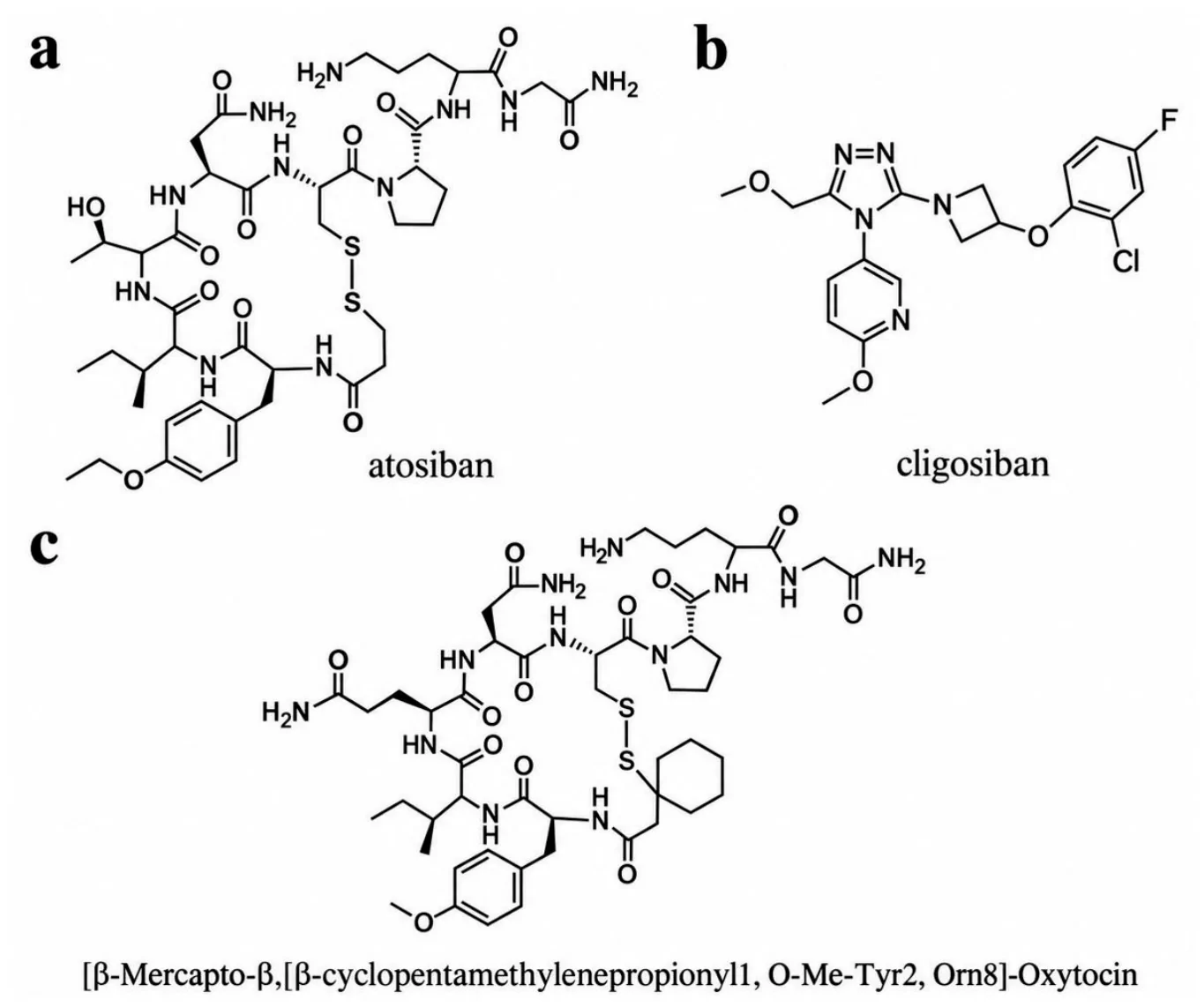
Molecular structures of (**a**) atosiban, (**b**) cligosiban, and (**c**) βMββC. Adapted from Stadler et al. [91], © 2020 Stadler et al., under the Creative Commons Attribution licence (CC BY). Selected structures were combined and relabelled.

Atosiban antagonises oxytocin and vasopressin receptor pathways, limiting attribution of responses specifically to OXTR (Figure 2a) [122,123]. Its established obstetric use [124,125,126,127] and tissue-dependent signalling [128] do not demonstrate urinary efficacy. Table 1 distinguishes human tissue mechanistic observations from animal tissue findings. These observations support investigation of OXTR antagonism as an experimental target; they do not establish atosiban as a clinically validated treatment for male LUTSs or OAB.

Atosiban reduced spontaneous contractile activity in human prostate tissue in vitro [22]. The human detrusor study discussed in Section 4.3 did not establish bladder antagonism [89]. In rat bladder, atosiban attenuated OT-induced contractions at 10 µM in an age-comparison study [129]. A subsequent obesity study reported reduced spontaneous activity in bladder and prostate tissue and reduced OT-induced bladder contractility in obese rats [130]. These are experimental contractile outcomes, not evidence of clinical urinary benefit.

#### 6.3.2. Cligosiban

Cligosiban is relatively selective for OXTR over vasopressin receptors (Figure 2b) [131]. Its evidence spans three distinct settings: rat bladder strips, a rat ejaculation model, and a clinical trial for premature ejaculation (Table 1). It reduced spontaneous bladder contraction frequency but did not significantly inhibit OT-induced contraction in young or older rats at tested concentrations [129]. Inhibition of induced ejaculation in rats [131] and the absence of significant benefit in the phase IIb premature-ejaculation trial [132] concern reproductive endpoints. Neither provides evidence of clinical efficacy for male LUTSs or OAB.

#### 6.3.3. Beta-Mercapto-Beta, Beta-Cyclopentamethylene-Propionyl

βMββC (Figure 2c) has preclinical evidence from isolated rat bladder strips and an in vivo model in anaesthetised female rats (Table 1). It attenuated OT-induced strip contractions at 1 µM in young and older rats [129]. In vivo, it attenuated the OT-evoked fall in intravesical pressure: −8.2 ± 0.6% after blockade versus −41.9 ± 2.9% in sham controls [133]. This supports receptor involvement in that model, not an independent pressure-lowering effect or symptom benefit. The urinary experiments do not establish selectivity over vasopressin receptors, and the different response directions underscore the importance of experimental setting. No presented evidence establishes clinical urinary efficacy.

### 6.4. Translational Priorities

Translation requires clinical phenotypes to be defined independently of experimental tissue responses. Histological BPH, BPE, BPO, OAB, and DO should be documented separately where assessed, using the distinctions outlined in Section 2 [28,29,102].

The translational question is whether selective OXTR modulation improves urinary symptoms or bladder function beyond isolated tissue contraction. Studies should distinguish symptom-defined male LUTSs or OAB from anatomical BPE, demonstrated BPO, and urodynamically observed DO. Outcomes should assess outlet resistance, storage function, voiding efficiency, and patient-reported benefit as appropriate. Ejaculatory outcomes are separate endpoints and cannot establish urinary efficacy [28,29,102,131,132].

The rat tissue findings summarised in Table 1 support further investigation of OXTR antagonism [129,130]. However, responses vary with the model, and inconsistent human detrusor responses limit extrapolation [89]. These findings do not demonstrate symptom improvement or relief of prostatic obstruction.

Further translation would require validated receptor localisation, receptor-selective concentration–response studies with vasopressin receptor counter-screening, and replication across age, sex, and metabolic status. Studies would need to determine whether tissue effects translate into improved storage or voiding without impaired emptying. Pharmacokinetics, target engagement, chronic safety, and endocrine or reproductive effects also require evaluation. Clinical urinary benefit remains a hypothesis to be tested rather than an expected consequence of reduced tissue contraction.

### 6.5. Potential Applications of Artificial Intelligence in Oxytocin Research

AI could assist OXTR research by quantifying compartment-specific receptor staining and contraction patterns, testing whether receptor localisation relates to antagonist responses. Existing clinical AI studies provide methodological context [134,135,136], rather than validation of this hypothesis. These proposed applications require independent validation and experimental confirmation; they currently provide no basis for OXTR-directed drug or patient selection.

## 7. Conclusions

This narrative review compares mechanistic findings, experimental settings, and reported responses to OXTR antagonists in prostate and bladder models. Comparable clinical efficacy data for atosiban, cligosiban, and βMββC in male LUTSs or OAB are unavailable in the reviewed evidence. Differences in receptor selectivity, species, tissue preparations, and endpoints preclude a therapeutic ranking.

Male LUTSs are multifactorial and should not routinely be attributed to prostate enlargement alone. Histological BPH, BPE, and BPO describe different pathological, anatomical, and functional findings; symptom-defined OAB and urodynamically observed DO are also distinct. OXTR expression and experimental modulation of prostate or bladder contractility support OXTR antagonism as a promising research target. They do not establish relief of BPO, improvement in OAB symptoms, or clinical efficacy for male LUTSs. OXTR antagonists should therefore be regarded as investigational for these indications, pending studies of clinical benefit, receptor selectivity, and safety.

## Figures and Tables

**Table 1:** Experimental findings and evidence maturity for OXTR antagonists. The categories describe study settings rather than formal evidence grades. The compounds were studied in different species, preparations, and endpoints, so the table does not compare or rank their clinical efficacy. Human tissue experiments are mechanistic studies, and the cligosiban clinical trial assessed premature ejaculation. None of the presented evidence establishes clinical efficacy for male LUTSs attributed to benign prostatic disease (“LUTS/BPH”) or symptom-defined OAB. OXTR, oxytocin receptor; OT, oxytocin; βMββC, β-mercapto-β,β-cyclopentamethylenepropionyl oxytocin analogue.

Compound	Receptor Selectivity	Experimental Model	Main Finding	Level of Evidence
Atosiban [22,89]	OXTR/vasopressin receptor antagonist; limited selectivity [122,123].	Human prostate and detrusor strips ex vivo.	Reduced spontaneous prostate activity; human detrusor study did not establish reliable OT contraction or antagonist inhibition.	Human tissue mechanistic evidence; no clinical urinary efficacy.
Atosiban [129,130]	As above.	Young/older and obese/non-obese rat bladder/prostate strips.	Reduced selected spontaneous responses; attenuated OT-induced bladder contraction, with age/metabolic context affecting responses.	Preclinical animal tissue evidence.
Cligosiban [129]	Selective OXTR antagonist relative to vasopressin receptors [131].	Young and older rat bladder strips.	Reduced spontaneous frequency; no significant inhibition of OT-induced contraction at tested concentrations.	Preclinical animal tissue evidence.
Cligosiban [131]	As above.	Rat apomorphine-induced ejaculation model.	Inhibited induced ejaculation; urinary outcomes not assessed.	Preclinical reproductive evidence.
Cligosiban [132]	As above.	Phase IIb placebo-controlled trial in men with lifelong premature ejaculation.	No significant benefit over placebo; LUTS/OAB outcomes not assessed.	Clinical evidence for a different indication; no clinical urinary efficacy.
βMββC [129]	Peptide OT antagonist; urinary studies do not establish selectivity over vasopressin receptors.	Young and older rat bladder strips.	Attenuated OT-induced contractions at 1 µM in both age groups.	Preclinical animal tissue evidence.
βMββC [133]	As above.	Anaesthetised female rat bladder in vivo.	Attenuated the OT-induced fall in intravesical pressure; does not demonstrate an independent pressure-lowering effect.	Preclinical animal physiological evidence.

## Data Availability

No new data were created or analyzed in this study. Data sharing is not applicable to this article.
